# *BRCA* testing in Asian ovarian cancer patients: Standard clinical practice or Mutation prediction model?

**DOI:** 10.1158/1055-9965.EPI-25-2008

**Published:** 2026-07-01

**Authors:** Boon Hong Ang, Sook-Yee Yoon, Joanna Lim, Nur Tiara Hassan, Mei Chee Tai, Zhi Lei Wong, Jo Yi Chow, Xin Wen Lee, Meow-Keong Thong, Gaik-Siew Ch’ng, Jamil Omar, Chee-Meng Yong, Ismail Aliyas, Rozita Abdul Malik, Suguna Subramaniam, Wee-Wee Sim, Chun Sen Lim, Saw-Joo Lee, Keng-Joo Lim, Mohamad Nasir Shafiee, Fuad Ismail, Mohd Pazudin Ismail, Suresh Kumarasamy, John Seng Hooi Low, Ahmad Muzamir Ahmad Mustafa, Mary J. Makanjang, Shahila Tayib, Nellie L.C. Cheah, Chee-Kin Fong, Kean-Fatt Ho, Azura Deniel, Soo-Fan Ang, Ahmad Radzi Ahmad Badruddin, Lye-Mun Tho, Yin Ling Woo, Weang-Kee Ho, Soo-Hwang Teo

**Affiliations:** 1 Cancer Research Malaysia, 1, Jalan SS12/1A, Subang Jaya, Malaysia; 2 School of Biosciences, Faculty of Science and Engineering, University of Nottingham, Semenyih, Malaysia; 3 Genetic Medicine Unit, University of Malaya Medical Center, Kuala Lumpur, Malaysia; 4 Genetics, Penang Hospital, Penang, Penang, Malaysia; 5 Genetics, Hospital Kuala Lumpur, Kuala Lumpur, Wilayah Persekutuan, Malaysia; 6 Gynaeoncology, Institut Kanser Negara, Putrajaya, Wilayah Persekutuan Putra, Malaysia; 7 Gynaeoncology, Hospital Ampang, Kuala Lumpur, Malaysia; 8 Gynaeoncology, Hospital Sultanah Bahiyah, Alor Setar, Kedah, Malaysia; 9 Clinical Oncology, University of Malaya, Kuala Lumpur, Wilayah Persekutuan, Malaysia; 10 Gynaeoncology, Hospital Wanita Dan Kanak-Kanak Sabah, Kota Kinabalu, Malaysia; 11 Gynaeoncology, Hospital Umum Sarawak, Kuching, Sarawak, Malaysia; 12 Gynaeoncology, Hospital Sultan Ismail, Johor Bharu, Johor, Malaysia; 13 Gynaeoncology, Hospital Raja Permaisuri Bainun, Ipoh, Perak, Malaysia; 14 Gynaeoncology, KPJ Johor Specialist Hospital, Johor, Malaysia; 15 Gynaeoncology, Hospital Universiti Kebangsaan Malaysia, Cheras, Kuala Lumpur, Malaysia; 16 Oncology, Universiti Kebangsaan Malaysia Medical Centre, Kuala Lumpur, Wilayah Persekutuan, Malaysia; 17 Gynaeoncology, Hospital Universiti Sains Malaysia, Kota Bahru, Kelantan, Malaysia; 18 Gynaeoncology, Gleneagles Penang, Penang, Malaysia; 19 Oncology, Pantai Hospital Kuala Lumpur, Kuala Lumpur, Wilayah Persekutuan, Malaysia; 20 Gynaeoncology, Hospital Tengku Ampuan Afzan, Kuantan, Pahang, Malaysia; 21 Gynaeoncology, KPJ Sabah Specialist Hospital, Kota Kinabalu, Sabah, Malaysia; 22 Gynaeoncology, Penang General Hospital, Georgetown, Pulau Pinang, Malaysia; 23 Oncology, Loh Guan Lye Specialist Centre, Penang, Malaysia; 24 Gynaeoncology, Subang Jaya Medical Centre, Subang Jaya, Malaysia; 25 Oncology, Mount Miriam Cancer Hospital, Tanjong Bungah, Penang, Malaysia; 26 Oncology, KPJ Ampang Puteri Specialist Hospital, Ampang, Kuala Lumpur, Malaysia; 27 Oncology, Penang Adventist Hospital, Penang, Penang, Malaysia; 28 Oncology, Beacon Hospital Sdn Bhd, Petaling Jaya, Malaysia; 29 Department of Obstetrics and Gynaecology, Faculty of Medicine, University of Malaya, Kuala Lumpur, Malaysia; 30 Department of Biomedical Science, Sunway University, 5, Jalan Universiti, Bandar Sunway, Selangor, Malaysia; 31 University Malaya Cancer Research Institute, Faculty of Medicine, University of Malaya, Kuala Lumpur, Malaysia

**Keywords:** *BRCA* testing, Ovarian cancer, Asian, Predictive model, Targeted testing

## Abstract

**Background:**

Germline *BRCA1/2* testing is recommended for all ovarian cancer patients, as identifying pathogenic variants (PVs) informs treatment and enables family cascade testing. However, in resource-limited settings, high testing costs often limit feasibility. An alternative approach is to use predictive models to prioritize patients at risk, optimizing resource allocation. Existing models are largely Western-derived; in Asian populations, models exist for breast cancer—not ovarian cancer.

**Methods:**

Using data from a multi-center study of 1,126 Asian patients with ovarian cancer (including 147 *BRCA* PV carriers), we developed models incorporating cancer history, clinicopathological features, and reproductive factors to estimate likelihood of carrying PVs. We assessed discrimination, calibration, and accuracy, and compared genetic testing costs to a universal testing strategy.

**Results:**

Our final model demonstrated good calibration and strong discriminatory power, with an area under the curve of 0.80 (95% confidence interval:0.74–0.87). Factors included in the model were age at diagnosis, ethnicity, personal and family cancer history, and clinicopathological features. The model had 77% accuracy at the optimal threshold for testing, compared to 13% accuracy with universal testing, reducing genetic testing costs from ~USD4,371 to ~USD1,974 per identified carrier. Notably, while maintaining 100% sensitivity, the model reduced testing by 15%, yielding potential savings of ~USD68,182 per 800 patients each year.

**Conclusion:**

Targeted testing using a prediction model potentially offers a more efficient and scalable alternative when universal testing is not feasible, optimizing impact in resource-limited settings.

**Impact:**

This work supports more affordable and equitable *BRCA* testing pathways for Asian ovarian cancer patients.

## Introduction

Genetic testing for germline *BRCA1* and *BRCA2* pathogenic variants (PVs) in patients with breast or ovarian cancer improves survival via targeted therapies such as PARP inhibitors (1). It also facilitates family cascade testing—often more cost-effective than treatment, especially in low-resource settings. Although ovarian cancer is less common than breast cancer, *BRCA* mutations are more prevalent in ovarian cancer, making systematic genetic testing essential to identify carriers and offer appropriate interventions (2). Up to 60% of carriers report no family history of cancer, so family history alone misses many *BRCA* PV carriers (2–4). Accordingly, guidelines such as the Mainstreaming Cancer Genetic Testing (MCG) (5) and the National Comprehensive Cancer Network (NCCN) (6) recommend universal testing for all ovarian cancer patients, irrespective of tumor subtype or grade (7–9). However, universal access to genetic testing remains challenging. In high-income countries, resources are available but underutilized by ethnic minorities (10) partly due to barriers such as lack of referrals, whereas the challenge in low- and middle-income countries (LMICs) is the lack of resources, including funding and manpower. Most services are funded by charitable or research funding and are largely inaccessible to the wider population (2,11). In such contexts, identifying individuals who have a higher probability of carrying a germline alteration, may be needed to balance cost and access.

*BRCA* carrier prediction models for breast cancer have been developed globally, including Malaysia (12–15). These models incorporate clinicopathological, demographic, and cancer history features to generate personalized risk scores, identifying individuals likely to carry *BRCA* PVs and prioritize them for genetic testing. We previously developed the Asian Genetic Risk Calculator (ARiCa) for breast cancer patients (15), calibrated to a *BRCA2*-predominant spectrum and breast-specific predictors (such as hormone receptor status and bilateral disease). Ovarian cancer, by contrast, exhibits a *BRCA1*-predominant spectrum and distinct epidemiology (such as histologic subtype and staging system), making ARiCa less suitable (2). Moreover, existing ovarian models are largely Western-derived—developed and validated primarily in high-risk groups—tend to underestimate carrier probabilities in Asian populations (12,13,16,17), underscoring the need for a cancer-specific model tailored to Asian women in the general population.

Hence, this study aims to develop and validate population-specific *BRCA* carrier prediction model for ovarian cancer patients in the general population, and to compare its performance to the current standard of universal testing. The findings can assist in guiding genetic testing decisions in low-resource settings, enabling a more efficient and equitable allocation of healthcare resources.

## Materials and Methods

### Study population

The study participants included women clinically diagnosed with ovarian, fallopian, or primary peritoneal cancer, recruited from two cohorts: [1] The Malaysian Ovarian Cancer Genetic (MyOvCa) study, a single-centre hospital-based case cohort where consecutive cases from a private hospital were recruited between October 2008 and February 2015 (3), and [2] The Mainstreaming Genetic Counselling for Ovarian Cancer Patients in Malaysia (MaGiC) study, a multi-center hospital-based case cohort, sourcing cases from 23 different sites, both private and public hospitals across Malaysia (2). Further details on patients and recruitment sites are provided in previous studies (2,3).

Participants donated a blood or saliva sample that was processed and stored, completed a questionnaire that included information on lifestyle and reproductive history for ovarian cancer, and provided written informed consent. Germline DNA was sequenced using an amplicon-based Hi-Plex-NGS method on an Illumina platform (California, USA), as described previously (3). All identified pathogenic and variants of uncertain significance were confirmed by Sanger sequencing. The recruitment and genetic studies were approved by the Ethics Committees of the Ministry of Health Malaysia (NMRR-16-1322-31114), University Malaya Medical Centre (UMMC 20163-2255), Hospital Universiti Sains Malaysia (USM/JEPeM/17060286), Universiti Kebangsaan Malaysia (JEP 2017 814), and Subang Jaya Medical Centre (RSDH 201612.2).

### Statistical analyses

#### Model development

Variables that were considered for the model development were age at diagnosis for ovarian cancer, ethnicity (Chinese, Malay, Indian, or Other), laterality (unilateral or bilateral), tumor grade, cancer stage, subtype, personal cancer history (breast, uterine, cervical, or colorectal cancers), and presence of first- and second-degree family history of breast or ovarian cancer. The grade index of carcinomas was determined based on the morphology of cancer cells observed from excised tumors under a microscope, as documented in histopathological reports. Grade 1 was assigned to well-differentiated carcinomas, grade 2 to moderately differentiated carcinomas, and grade 3 to poorly differentiated carcinomas. Cancer stage was assigned based on the International Federation of Gynecology and Obstetrics (FIGO) system, which is determined by the extent of spread and metastasis. Cancer subtype analyzed in present study were serous and non-serous subtypes encompassing endometroid, clear cell, mucinous, mixed, adenocarcinoma, and rare/unclassified. Both grade and subtype were further grouped together according to criteria established in previous publications: [1] Subtype-Grade v1 - high-grade serous, low-grade serous, endometrioid, clear cell, mucinous, and other (mixed, adenocarcinoma, and rare/unclassified) (18), [2] Subtype-Grade v2 - high-grade serous, high-grade endometrioid, high-grade clear cell, and other (low-grade serous, low- grade endometrioid, low-grade clear cell, mucinous, mixed, adenocarcinoma, and rare/unclassified) (19). Additionally, hormonal use and reproductive factors considered were oral contraceptive use (ever or never), age at menarche, menopausal status (pre-menopause or post-menopause), parity status (parous or nulliparous), and history of tubal ligation (ever or never).

The study sample was split into training (70%) and validation (30%) sets using stratified random sampling by carrier status to preserve mutation prevalence (13%) in both datasets; all other variables were split randomly. Equality tests indicated broadly similar distributions of key variables and missingness between the two datasets (Supplementary Table S1). Laterality, tumor grade, and cancer stage had missing rates exceeding 10%: 27% for laterality, 33% for grade, and 17% for stage (Table 1). Missing data in both the training and validation sets were imputed using multiple imputation by chained equations, assuming missing at random (MAR). Personal history of other cancers and *BRCA* PV carrier status were included in the imputation model, as they have demonstrated importance as predictors of the variables in this study. We assessed the plausibility of the MAR assumption by fitting logistic regression models for the missingness indicator of each variable against observed covariates. Missingness was associated with at least one observed variable included in the imputation model, suggesting that missingness was related to observed information and supporting multiple imputation under MAR assumption. We generated 100 imputed datasets for the analysis, with each imputed dataset analyzed separately and then combined according to Rubin’s rules (20).

*BRCA* carrier prediction models were developed using logistic regression on the training set. Candidate predictors entered the multivariable model if they met a liberal univariable threshold (p-value<0.20) to avoid excluding potentially informative variables; retention in the final model used a stricter criterion (p-value<0.05). A correlation test (r) was performed to assess multicollinearity among variables. For highly correlated variables, the one with the higher odds ratio (OR) was prioritized for inclusion. Therefore, menopausal status, parity status, and presence of family cancer history were retained, while age at menopause, number of children, age at diagnosis and number of affected relatives were excluded due to their strong correlation with the retained variables (r=0.696, r=0.835, and r>0.999, respectively).

In principle, variables with p-value≥0.05 were excluded from the final models; however, we retained key predictors such as age at diagnosis, personal and family cancer history, and tumor characteristics irrespective of their p-values in the gene-specific and overall *BRCA* models, given their well-established importance in the literature (3,4,21). Oral contraceptive use and menopausal status were also retained because they improved discrimination between carriers and non-carriers. Hormonal use has been reported to be less common among carriers diagnosed with ovarian cancer, while menopausal status captures gene-specific differences in age at diagnosis, particularly the later onset observed in *BRCA2* PV carriers (22). Recognizing that certain variables may not be relevant for specific mutation types, we explored eight different model combinations of candidate predictors in this study: [1] a baseline model encompassing demographics, personal, and family cancer history components (Model 1), [2] a reproductive model that adds oral contraceptive use, menopausal status, and parity to the baseline (Model 2), [3] three subtype models that add tumor grade and subtype to the baseline, specified as Model 3 (grade and subtype entered separately), Model 4 (Subtype–Grade v1), and Model 5 (Subtype–Grade v2); and [4] three full models combining the reproductive model with tumor grade and subtype, specified as Model 6 (grade and subtype entered separately), Model 7 (Subtype–Grade v1), and Model 8 (Subtype–Grade v2). Accordingly, we developed an overall *BRCA* model as well as *BRCA*-specific models tailored specifically for *BRCA1* and *BRCA2*. In total, we have developed 24 models: 8 models for overall BRCA prediction and 16 *BRCA*-specific models (8 for *BRCA1* and 8 for *BRCA2*).

#### Validation of performance of risk assessment methods

Model discrimination and calibration were assessed in the validation set. Discrimination was evaluated using the area under the receiver operating curve (AUC) (23). Calibration was assessed using the Hosmer–Lemeshow (HL) test (HL, 15 or less) and calibration plots to visualize the distribution of observed versus expected numbers of *BRCA* PV carriers across deciles of predicted carrier probabilities (24). The optimal carrier probability threshold for genetic testing was chosen based on the intersection of sensitivity and specificity curves (25). Additional performance measures included sensitivity (percentage of true carriers detected), specificity (percentage of true non-carriers detected), accuracy (percentage of true carriers and non-carriers detected), and screening rate (percentage of eligible patients for *BRCA* testing).

We conducted a simple cost analysis using a cost-threshold scenario (base-case ~USD570 per test) and sensitivity analyses at reduced unit test cost (~USD115 per test) and varying subsidy levels. Efficiency was assessed using detection and cost ratios. The detection ratio is the number of tests needed to find one *BRCA* PV carrier; the cost ratio is the testing cost per carrier detected. We estimated total testing costs and cost per carrier using the annual incidence (~800 cases) and mutation prevalence (13%), assuming 100% compliance in low-risk groups. All data were analyzed using R version 4.0.3 (RRID:SCR_001905), and a p-value<0.05 was considered statistically significant unless otherwise stated.

## Results

In this cross-sectional multi-center case study of 1,126 patients with ovarian cancer, 147 (13.0%) had a germline *BRCA* PV, with 9% *BRCA1* and 4% *BRCA2* PV carriers (Table 1). The majority were Chinese (44%) and Malay (40%), with a mean age at diagnosis of 51.8 years (standard deviation=11.3). Indian, Chinese, and Malay women had higher proportions of advanced-stage (classified as stage 3 and 4) disease (61.0%, 58.5%, and 56.7%, respectively), compared to Other ethnic group (46.3%; p-value=0.004). Additionally, Indian and Malay women had higher proportions of carriers compared to Chinese women (16.5% and 16.7% versus 9.4%, respectively; p-value=0.043). Compared to non-carriers, both *BRCA1* and *BRCA2* PV carriers are more likely to have serous epithelial ovarian cancers, present at later stages with higher grades (classified as grade 2 and 3), and report a personal history of breast cancer (p-value<0.05; Supplementary Table S2). A higher proportion of *BRCA* PV carriers reported a first- or second-degree relative with breast and/or ovarian cancer (p-value<0.05), but more than half had no family history. While these trends hold true for both *BRCA1* and *BRCA2* PV carriers, *BRCA2* PV carriers were more likely to be post-menopausal or parous (p-value<0.05). Conversely, *BRCA1* PV carriers were more likely to have a family history of ovarian cancer compared to *BRCA2* PV carriers (p-value<0.05). Due to these differences, we decided to explore separate models for *BRCA1, BRCA2*, and overall *BRCA* in subsequent analyses.

Prediction models were developed using 788 ovarian cancer cases (103 *BRCA* PV carriers) and validated using 338 cases (44 *BRCA* PV carriers; Supplementary Fig. S1). In univariable analysis, family history of breast cancer, type of cancer, personal history of breast cancer, tumor grade, cancer stage, and subtype were associated with overall *BRCA, BRCA1*, and *BRCA2* PV carrier status (p-value<0.2; Supplementary Table S3). Age at diagnosis, ethnicity, laterality, personal history of colorectal cancer, menopausal status, and parity were associated only with *BRCA2* PV carrier status, whereas family history of ovarian cancer was associated exclusively with *BRCA1* PV carrier status (p-value<0.2).

**Figure 1:**
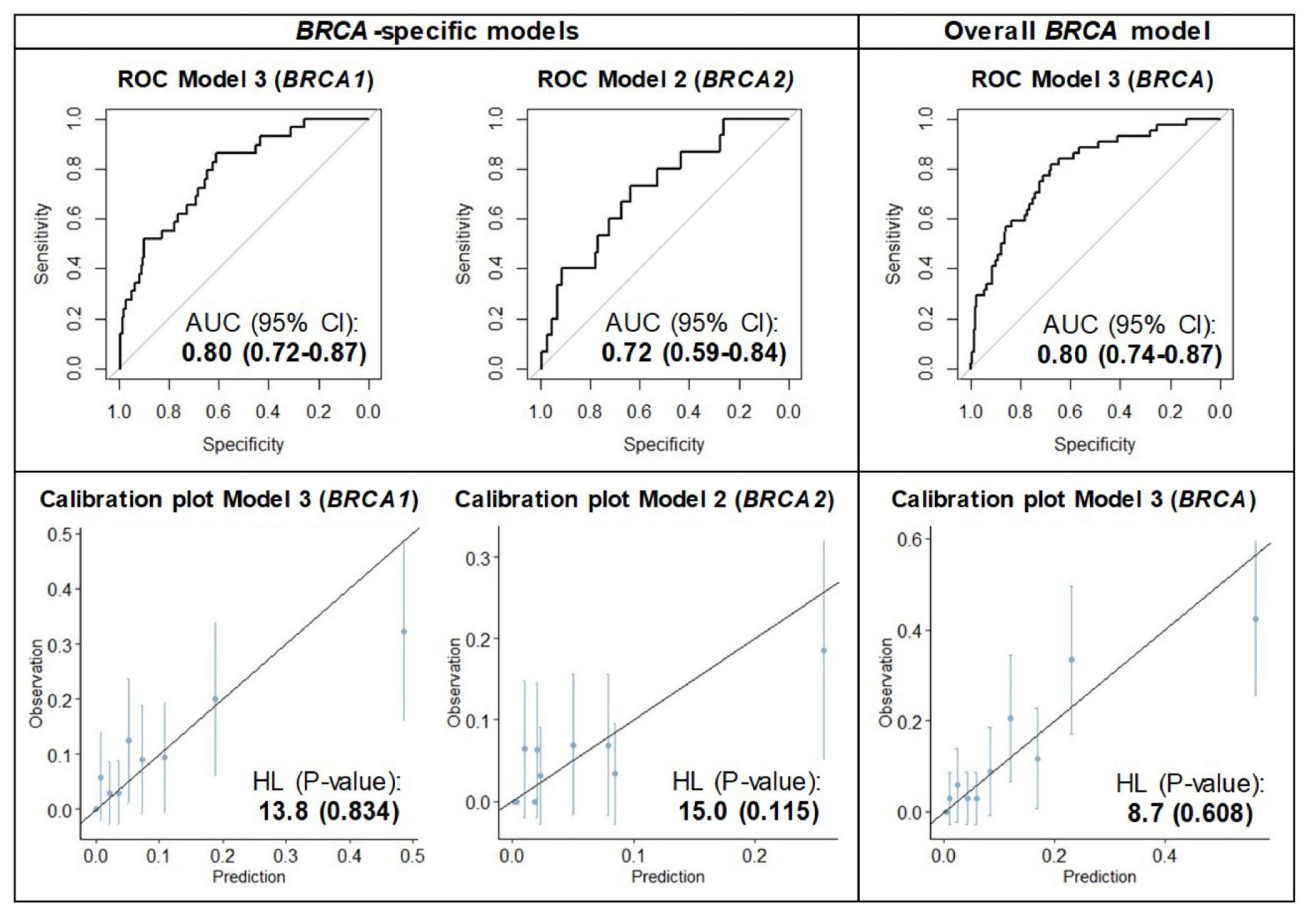
Receiver operating curve and calibration plot of best-performing models by *BRCA* PV carrier status Sample: 338 ovarian cancer patients from the Malaysian Ovarian Cancer Genetic (MyOvCa) study and the Mainstreaming Genetic Counselling for Ovarian Cancer Patients in Malaysia (MaGiC) study in imputed validation set. Abbreviations: AUC, Area Under Curve; 95% CI, 95% Confidence Interval; HL, Hosmer-Lemeshow; ROC, Receiver Operating Curve. Note: Variables included in BRCA-specific models (Model 2 and 3): Age of diagnosis, ethnicity, oral contraceptive use, menopausal status, parity status, family history of breast or ovarian cancer (first and second degree), personal history of cancer (ovarian, breast, or colorectal cancer), tumor grade, cancer stage and subtype. Variables included in Overall BRCA model (Model 3): Age of diagnosis, ethnicity, family history of breast or ovarian cancer (first and second degree), personal history of cancer (ovarian, breast, or colorectal cancer), laterality, tumor grade, cancer stage and subtype.

The best-performing model was selected based on the highest AUC and the lowest non-significant HL score in the validation set (Supplementary Table S4). Among the eight models, subtype models (Model 3, 4, and 5) were the best-performing models for overall *BRCA* and *BRCA1*, whereas reproductive model (Model 2) for *BRCA2*. Even though the full models (Model 6, 7, and 8) have comparatively high AUCs, addition of hormonal use and reproductive components to the subtype models did not improve AUCs of the model performance for overall *BRCA, BRCA1*, and *BRCA2*. Subtype models with grade and subtype as individual variables (Model 3) performed better in terms of calibration compared to those incorporating the combined versions of grade and subtype components (Model 4 and 5). For *BRCA2*, addition of tumor characteristics (Model 3-8) reduces the discriminatory power, addition of hormonal use and reproductive components to the baseline model (Model 2), on the other hand improves discrimination while maintaining good calibration. Therefore, Model 3 was the best-performing model for overall *BRCA* (AUC=0.80, HL=13.8) and *BRCA1* (AUC=0.80, HL=8.7), whereas Model 2 performed best for *BRCA2* (AUC=0.72, HL=15.0; Figure 1). Overall, the AUCs were comparable across models.

Table 2 presents the final set of variables included in each model and summarizes the corresponding adjusted estimates. Malay ethnicity, personal history of breast cancer, advanced cancer stage, presence of first- and second-degree family history of breast cancer or ovarian cancer, and clear cell subtype were associated with overall *BRCA* PV carrier status (p-value<0.05). These variables were also associated with *BRCA1* PV carrier status, except for second-degree family history of breast cancer, whereas *BRCA2* was only associated with Malay ethnicity, personal history of breast cancer, first-degree family history of breast cancer, and parity status (p-value<0.05).

We identified an optimal testing threshold for each model, with the overall *BRCA* model requiring a higher threshold (Supplementary Fig. S2). At these thresholds, we estimated the proportion of ovarian cancer patients who would be offered *BRCA* testing and compared performance (Table 3). Overall *BRCA* model would screen 20% fewer patients (33% v 53%) to detect 5% more carriers compared to *BRCA*-specific models (73% v 68%). We then compared the overall *BRCA* model with universal testing. Table 4 showed that, at the optimal threshold, the model achieved higher accuracy while screening 67% fewer patients and costing three times less than universal testing, successfully capturing over 70% of carriers. Notably, achieving 100% sensitivity with the model would require screening 85% of ovarian cancer patients, which is still more efficient than the current guideline recommending testing of all affected individuals.

**Figure 2:**
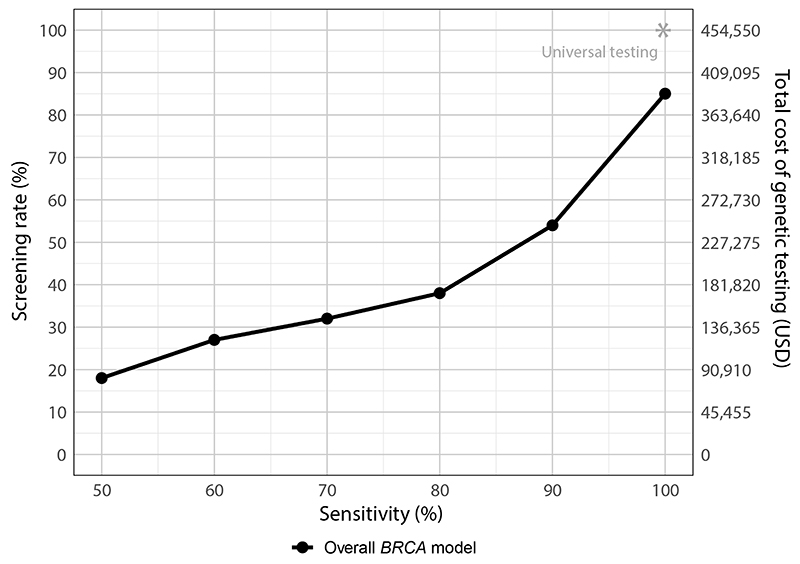
Screening rate and the corresponding total cost of genetic testing based on annual ovarian cancer incidence at varying sensitivity under base-case unit test cost Sample: 338 ovarian cancer patients from the Malaysian Ovarian Cancer Genetic (MyOvCa) study and the Mainstreaming Genetic Counselling for Ovarian Cancer Patients in Malaysia (MaGiC) study in imputed validation set. Note: Total cost of genetic testing (~USD570 per test) was calculated based on country-specific annual incidence (800 cases), assuming 100% uptake and compliance in high-risk and low-risk groups, respectively.

Additionally, we presented a cost-threshold scenario comparing the total cost of genetic testing at different sensitivity levels, assuming an ideal scenario of 100% uptake among high-risk individuals and 100% compliance among low-risk individuals. Figure 2 illustrates that the model was more cost-effective, screening 15% fewer patients and saving up to ~USD68,182 (~MYR 300,000) while achieving 100% sensitivity—comparable to universal testing. When the unit cost was reduced from ~USD570 to ~USD115 (Supplementary Fig. S3), our conclusions were unchanged: the estimated 15% reduction in testing volume persisted, while affordability improved despite smaller absolute savings. To examine cost variation, we plotted total costs across different subsidy levels and corresponding screening rates, adjusted for observed uptake among ovarian cancer patients from a recent study (26). Total costs remained consistently lower for the overall *BRCA* model at every subsidy level (Supplementary Fig. S4).

## Discussion

### Summary of main results

Despite the importance of germline *BRCA1* or *BRCA2* PVs testing for risk management and treatment selection, genetic testing uptake remains low in Asia due to resource limitations. An efficient mutation prediction model is therefore crucial for improving detection rates, identifying individuals who would benefit most from testing, and optimizing resource allocation. We demonstrated that our proposed model for ovarian cancer patients achieves 100% sensitivity—matching universal testing—while reducing the number of patients screened by 15%, offering a more resource-efficient approach to capture all carriers.

### Results in the context of published literature

The clinical features and risk factors seen in women with *BRCA* mutations in this study are similar to those reported in other Asian studies and partly overlap with patterns seen in breast cancer (3,15,21,27–30). While tumor grade is a key predictor for *BRCA* mutation in breast cancer (15), ovarian cancer’s aggressive nature and the predominance of high-grade tumors (90% classified as grade 3 in present study) limit the discriminatory power of grade (31). Breast cancer typically progresses more slowly and has a higher 5-year survival rate, with tumor grades varying widely across early and late stages (32). In contrast, ovarian cancer is inherently aggressive, often presenting as high-grade from the outset and associated with lower survival rates (32). As a result, cancer stage—which better reflects disease progression and prognosis—emerged as a more informative predictor for *BRCA* mutation in ovarian cancer, underscoring the importance of a cancer-specific model that accounts for key factors influencing *BRCA* mutation risk.

### Comparison with current practice in Western setting

Current *BRCA* testing approaches in the West typically follow either universal testing (based on NCCN guideline) for epithelial ovarian cancer or criteria-driven referral based on mutation prediction models (e.g., BRCAPRO and CanRisk) (33–36). However, these models rely heavily on detailed pedigree information, including age at diagnosis across multiple relatives, to estimate carrier probability and apply a fixed referral threshold (≥5% based on NCCN guideline) (36). In contrast, our models differ in both structure and intended use. Rather than relying primarily on extended family history, it incorporates routinely available clinical and tumor characteristics, allowing implementation in settings where pedigree information is often incomplete or unavailable. Notably, in our cohort, the threshold that maximized sensitivity and specificity was 12.2%, which is higher than the recommended 5% referral threshold in Western settings; applying a 5% threshold in our cohort would result in substantially broader testing with limited additional gain in sensitivity, highlighting the need for context-specific calibration of referral thresholds.

### Implications for practice and future research

Despite international recommendations to test all ovarian cancer patients, implementation gaps persist in many Asian settings: in Singapore, only 18.1% of eligible ovarian cancer patients were referred to a cancer genetics clinic, and in Malaysia only ~17% proceeded with testing when self-funded (26,37). These gaps likely reflect persistent barriers in routine care, including high testing costs and limited clinical genetics services (2,7,10,11,37). Consistent with this, a local study showed that genetic testing uptake among ovarian cancer patients is influenced by the subsidy levels, with higher uptake observed when testing is fully subsidized (26). In LMICs, constraints extend beyond laboratory price alone to include counselling resources, referral logistics, and overall service delivery capacity. A strategy that preserves sensitivity while reducing testing volume may therefore improve feasibility and scalability. Previously, we showed that targeted *BRCA* testing using a *BRCA* carrier prediction model spares low-risk individuals from unnecessary testing—an approach relevant to low-resource settings (15). Building on our prior work, our current study demonstrated that the proposed *BRCA* carrier prediction model for ovarian cancer supports a scalable referral-threshold strategy that can be adapted to available resources. In our data, a 1% threshold achieved 100% sensitivity while recommending testing for 85% of patients, whereas a 12.2% threshold recommended testing for 33% with 73% sensitivity, illustrating how programs can choose a cutoff that balances missed carriers against feasible testing volume and cost. In practice, as capacity and budgets increase, the threshold can be lowered to expand testing; when resources are constrained, it can be raised to focus testing on those at highest risk.

Although *BRCA* testing is often associated with targeted therapies and improved survival (38), its greatest impact in low-resource settings is enabling risk management and cancer prevention. By identifying *BRCA* PV carriers, we can implement preventive measures not only for affected individuals but also for their at-risk relatives. A systematic review found that *BRCA* cascade screening—involving the testing of both affected individuals and their unaffected relatives—is a cost-effective approach (39). A study conducted in a middle-income Asian country further demonstrated that familial cascade testing can be cost-saving (40). Our proposed mutation prediction model supports this preventive approach by prioritizing cancer patients most likely to carry *BRCA* PVs, thereby facilitating more targeted and efficient cascade testing in resource-constrained environments.

### Study limitations and strengths

This study is limited by a modest sample size and few carriers, increasing the risk of overfitting—especially in the gene-specific models. This reflects the rarity of ovarian cancer and the challenges of referring patients for genetic counselling and testing across geographically dispersed hospitals (2). While *BRCA1/2* prevalence appears broadly similar across Asian populations, underlying genetic architecture and baseline risk profiles may still vary across East Asian, South Asian, and other Asian populations, which could affect model performance and the optimal referral threshold. Our findings should therefore be viewed as exploratory, and external validation in larger, more diverse Asian cohorts—potentially with re-calibration—is needed before broader generalization or use in different settings. We did not include Ashkenazi Jewish ancestry in the model, unlike most existing models, as such variants are extremely rare in Malaysia. Moreover, self-reported ethnicity has been shown in prior studies to be highly concordant with genetic ancestry, making it a reliable measure for our population (41). Our model focused exclusively on *BRCA* genes because the number of carriers of other susceptibility genes was too small for meaningful analysis. Future work should expand the gene panel and consider incorporating homologous recombination deficiency (HRD) testing for broader risk assessment. We acknowledge that univariable selection of candidate predictors does not capture interactions; however, the included predictors were not highly correlated. Comparison with existing models (e.g., BRCAPRO and CanRisk) was not undertaken because they typically require detailed multigenerational pedigree/family-history inputs that were not consistently available in our cohort, whereas our model uses routinely collected clinical and tumor predictors. Lastly, this base-case cost analysis reflects a cost-threshold scenario rather than a full health economic evaluation, excludes cascade testing and downstream costs that warrant further decision modelling, and the relative advantage of targeted testing will depend on future cost trajectories and health system capacity. Nonetheless, this is the first Asian multi-center study with comprehensive data on clinicopathological and demographic features, marking an important step toward developing a cancer-specific *BRCA* carrier prediction model tailored for an unselected population and assessing its efficiency in a low-resource setting.

## Conclusions

Our study raises important questions about the effectiveness and feasibility of a cancer-specific *BRCA* carrier prediction model compared to universal testing. Given the substantial need for genetic testing, a tailored approach that takes into account available subsidies and healthcare capacity is essential. In resource-limited settings, our model improves efficiency by minimizing unnecessary testing and prioritizing high-risk individuals, while providing a scalable pathway to broader testing by adjusting referral thresholds to match local resource capacity and genetic testing costs.

## Supplementary Material

Fig. S1

Fig. S2

Fig. S3

Fig. S4

Table S1

Table S2

Table S3

Table S4

## Figures and Tables

**Table 1:** Characteristics of study population

Variable	Total n (%) (n=1,126)	Missing* n (%)	Chinese n (%) (n=489)	Malay n (%) (n=450)	Indian n (%) (n=115)	Other n (%) (n=68)	P-value
**Demographic**							
**Age at diagnosis**, *mean (sd)*	51.84 (11.3)	9 (0.8)	52.94 (11.14)	50.31 (11.2)	54.4 (11.8)	49.6 (11.3)	**<0.001**
**Ethnicity**, *n (%)*		4 (0.4)					
Chinese	489 (43.6)		-	-	-	-	
Malay	450 (40.1)		-	-	-	-	
Indian	115 (10.2)		-	-	-	-	
Other	68 (6.1)		-	-	-	-	
**Hormonal use and reproductive history**							
**Oral contraceptive**, *n (%)*		14 (1.2)					**0.017**
Never	872 (78.4)		393 (81.4)	332 (74.4)	97 (84.3)	49 (73.1)	
Ever	240 (21.6)		90 (18.6)	114 (25.6)	18 (15.7)	18 (26.9)	
**Age at menarche**, *mean (sd)*	13.02 (1.52)	18 (1.6)	13.12 (1.6)	12.92 (1.5)	12.99 (1.5)	13 (1.6)	0.240
**Menopausal status**, *n (%)*		19 (1.7)					**0.009**
Pre-menopause	213 (19.2)		88 (18.5)	89 (19.9)	14 (12.2)	22 (32.4)	
Post-menopause	894 (80.8)		388 (81.5)	358 (80.1)	101 (87.8)	46 (67.6)	
**Parity status**, *n (%)*		8 (0.7)					0.303
Nulliparous	367 (32.8)		160 (33.0)	155 (34.5)	29 (25.2)	23 (33.8)	
Parous	751 (67.2)		325 (67.0)	294 (65.5)	86 (74.8)	45 (66.2)	
**Tubal Ligation**, *n (%)*		53 (4.7)					0.277
Never	963 (89.7)		417 (88.7)	389 (91.3)	98 (86.7)	59 (93.7)	
Ever	110 (10.3)		53 (11.3)	37 (8.7)	15 (13.3)	4 (6.3)	
**Family history**							
**FFHBC**, *n (%)*		13 (1.2)					0.881
No	1009 (90.7)		433 (89.8)	409 (91.3)	105 (91.3)	61 (91.0)	
Yes	104 (9.3)		49 (10.2)	39 (8.7)	10 (8.7)	6 (9.0)	
**FFHOC**, *n (%)*		15 (1.3)					0.989
No	1058 (95.2)		459 (95.4)	425 (95.1)	109 (94.8)	64 (95.5)	
Yes	53 (4.8)		22 (4.6)	22 (4.9)	6 (5.2)	3 (4.5)	
**SFHBC**, *n (%)*		13 (1.2)					0.632
No	1027 (92.3)		449 (93.2)	408 (91.1)	106 (92.2)	63 (94.0)	
Yes	86 (7.7)		33 (6.8)	40 (8.9)	9 (7.8)	4 (6.0)	
**SFHOC**, *n (%)*		14 (1.2)					0.418
No	1094 (98.4)		471 (97.9)	444 (99.1)	112 (97.4)	66 (98.5)	
Yes	18 (1.6)		10 (2.1)	4 (0.9)	3 (2.6)	1 (1.5)	
**Personal history**							
**Type of cancer**, *n (%)*	7 (0.6)						0.728
Ovarian	1066 (95.3)		465 (95.1)	425 (94.4)	108 (93.9)	67 (98.5)	
Fallopian tube	20 (1.8)		7 (1.4)	11 (2.4)	3 (2.6)	0 (0.0)	
Peritoneal	32 (2.9)		14 (2.9)	13 (2.9)	4 (3.5)	1 (1.5)	
**Other cancer**, *n (%)*		0 (0.0)					
Breast cancer	48 (66.8)		27 (71.1)	13 (59.1)	6 (66.7)	2 (50.0)	0.211
Uterine cancer	14 (19.4)		5 (13.1)	6 (27.3)	2 (22.2)	2 (50.0)	0.557
Cervical cancer	5 (6.9)		3 (7.9)	1 (4.5)	1 (11.1)	0 (0.0)	0.662
Colorectal cancer	5 (6.9)		3 (7.9)	2 (9.1)	0 (0.0)	0 (0.0)	0.770
**Tumor characteristics**							
**Laterality**, *n (%)*		308 (27.4)					0.825
Unilateral	524 (64.1)		208 (62.5)	228 (64.6)	50 (64.9)	37 (68.5)	
Bilateral	294 (35.9)		125 (37.5)	125 (35.4)	27 (35.1)	17 (31.5)	
**Grade**, *n (%)*		372 (33.0)					0.064
Grade 1	78 (10.3)		19 (6.7)	47 (13.6)	4 (6.2)	8 (13.8)	
Grade 2	4 (0.6)		2 (0.7)	1 (0.3)	1 (1.5)	0 (0.0)	
Grade 3	672 (89.1)		263 (92.6)	298 (86.1)	60 (92.3)	50 (86.2)	
**Stage**, *n (%)*		186 (16.5)					**0.004**
Stage 1	274 (29.2)		106 (27.3)	125 (31.4)	28 (28.0)	15 (27.8)	
Stage 2	128 (13.6)		62 (16.0)	40 (10.1)	11 (11.0)	14 (25.9)	
Stage 3	438 (46.6)		187 (48.2)	177 (44.6)	55 (55.0)	19 (35.2)	
Stage 4	100 (10.6)		33 (8.5)	55 (13.9)	6 (6.0)	6 (11.1)	
**Subtype**, *n (%)*		33 (2.9)					0.193
Serous	565 (51.6)		241 (51.3)	218 (49.3)	67 (59.3)	39 (58.2)	
Endometrioid	225 (20.6)		85 (18.1)	99 (22.4)	28 (24.8)	13 (19.4)	
Clear cell	184 (16.8)		90 (19.1)	74 (16.7)	9 (8.0)	10 (14.9)	
Mucinous	40 (3.7)		17 (3.6)	19 (4.3)	4 (3.5)	0 (0.0)	
Mixed	25 (2.3)		10 (2.1)	14 (3.2)	0 (0.0)	1 (1.5)	
Adenocarcinoma	14 (1.3)		5 (1.1)	6 (1.4)	2 (1.8)	1 (1.5)	
Rare/Unclassified	40 (3.7)		22 (4.7)	12 (2.7)	3 (2.6)	3 (4.5)	
**Outcome**							
***BRCA* PVs carrier status**, *n (%)*		0 (0.0)					**0.043**
Non-carrier	979 (86.9)		443 (90.6)	375 (83.3)	96 (83.5)	61 (85.9)	
*BRCA1*	97 (8.6)		32 (6.5)	49 (10.9)	12 (10.4)	7 (9.9)	
*BRCA2*	50 (4.5)		14 (2.9)	26 (5.8)	7 (6.1)	3 (4.2)	

Sample:1,126 ovarian cancer patients from the Malaysian Ovarian Cancer Genetic (OVC) study and the Mainstreaming Genetic Counselling for Ovarian Cancer Patients in Malaysia (MaGiC) study before imputation. Abbreviations: FFHBC, First Degree Family History for Breast Cancer; FFHOC, First Degree Family History for Ovarian Cancer; SFHBC, Second Degree Family History for Breast Cancer; SFHOC, Second Degree Family History for Ovarian Cancer; PV, pathogenic variant.

**Table 2:** Multivariable regression of best-performing models by *BRCA* PV carrier status

Model	*BRCA*-specific models								Overall *BRCA* model			
	Model 3				Model 2				Model 3			
Variable	*BRCA1* versus Non-carrier (n=753)				*BRCA2* versus Non-carrier (n=720)				*BRCA* versus Non-carrier (n=788)			
	OR	95% CI		P-value	OR	95% CI		P-value	OR	95% CI		P-value
**Demographic**												
**Age at diagnosis**	0.97	0.94	1.00	0.089	0.99	0.96	1.04	0.917	0.99	0.96	1.01	0.261
**Ethnicity**												
Chinese	*Reference*				*Reference*				*Reference*			
Malay	2.52	1.23	5.17	**0.012**	4.20	1.64	10.77	**0.003**	3.09	1.70	5.61	**<0.001**
Indian	1.61	0.56	4.63	0.375	2.85	0.80	10.20	0.108	2.06	0.87	4.86	0.100
Other	0.49	0.10	2.54	0.397	1.26	0.14	11.17	0.836	0.62	0.16	2.36	0.482
**Hormonal use and reproductive history**												
**Oral contraceptive**												
Never					*Reference*							
Ever	-	-	-	-	1.06	0.47	2.43	0.881	-	-	-	-
**Menopausal status**												
Pre-menopause					*Reference*							
Post-menopause	-	-	-	-	8.17	0.86	77.64	0.068	-	-	-	-
**Parity status**												
Nulliparous					*Reference*							
Parous	-	-	-	-	5.27	1.49	18.63	**0.010**	-	-	-	-
**Family history**												
**Family history of cancer**	*Reference (No)*				*Reference (No)*				*Reference (No)*			
FFHBC (yes)	6.46	2.94	14.21	**<0.001**	5.52	2.29	13.33	**<0.001**	5.84	3.05	11.16	**<0.001**
FFHOC (yes)	7.64	3.06	19.05	**<0.001**	2.39	0.56	10.16	0.239	5.17	2.26	11.80	**<0.001**
SFHBC (yes)	1.94	0.71	5.28	0.197	1.95	0.62	6.18	0.254	2.35	1.03	5.37	**0.042**
SFHOC (yes)	17.75	3.28	95.98	**0.001**	-	-	-	-	7.88	1.69	36.80	**0.009**
**Personal history**												
**Type of cancer**												
Ovarian	*Reference*								*Reference*			
Fallopian tube	2.74	0.59	12.68	0.196	-	-	-	-	1.68	0.47	6.00	0.427
Peritoneal	0.15	0.02	1.42	0.098	-	-	-	-	0.19	0.04	0.90	**0.036**
**Other cancer**	*Reference (No)*				*Reference (No)*				*Reference (No)*			
Breast cancer (yes)	7.13	2.31	21.98	**0.001**	4.78	1.31	17.42	**0.018**	4.96	1.94	12.70	**<0.001**
Colorectal cancer (yes)	-	-	-	-	5.94	0.40	88.76	0.196	2.86	0.17	49.05	0.469
**Tumor characteristics**												
**Laterality**												
Unilateral	-	-	-	-	-	-	-	-	*Reference*			
Bilateral	-	-	-	-	-	-	-	-	1.72	0.91	3.26	0.093
**Grade**												
Grade 1-2	*Reference*				-	-	-	-	*Reference*			
Grade 3	4.90	0.68	35.13	0.113	-	-	-	-	3.33	0.84	13.17	0.087
**Stage**												
Stage 1	*Reference*								*Reference*			
Stage 2	6.31	1.34	29.65	**0.020**	-	-	-	-	2.92	0.85	10.06	0.090
Stage 3	4.27	0.36	10.61	**0.041**	-	-	-	-	4.78	1.65	13.89	**0.004**
Stage 4	9.43	0.69	14.68	**0.004**	-	-	-	-	5.77	1.77	18.77	**0.004**
**Subtype**												
Other ^a^	*Reference*								*Reference*			
Mucinous	2.56	0.12	53.88	0.545	-	-	-	-	0.81	0.06	10.66	0.871
Clear cell	-	-	-	-	-	-	-	-	0.14	0.02	0.90	**0.038**
Endometrioid	1.94	0.40	11.83	0.443	-	-	-	-	1.12	0.33	3.83	0.861
Serous	3.19	0.74	15.07	0.136	-	-	-	-	2.06	0.70	6.08	0.188

Sample: 788 ovarian cancer patients from the Malaysian Ovarian Cancer Genetic (MyOvCa) study and the Mainstreaming Genetic Counselling for Ovarian Cancer Patients in Malaysia (MaGiC) study in imputed training set.

Abbreviations: OR, Odds ratio; 95% CI, 95% Confidence Interval; FFHBC, First Degree Family History for Breast Cancer; FFHOC, First Degree Family History for Ovarian Cancer; SFHBC, Second Degree Family History for Breast Cancer; SFHOC, Second Degree Family History of Ovarian Cancer.

a Includes mixed, adenocarcinoma, rare, and unclassified.

Note: All variables listed in this table were included in the corresponding final models. Variables included in BRCA-specific models (Model 2 and 3): Age of diagnosis, ethnicity, oral contraceptive use, menopausal status, parity status, family history of breast or ovarian cancer (first and second degree), personal history of cancer (ovarian, breast, or colorectal cancer), tumor grade, cancer stage and subtype. Variables included in Overall BRCA model (Model 3): Age of diagnosis, ethnicity, family history of breast or ovarian cancer (first and second degree), personal history of cancer (ovarian, breast, or colorectal cancer), laterality, tumor grade, cancer stage and subtype.

**Table 3:** Performance of best performing models at optimal thresholds

Model	*BRCA-specific* models		Overall *BRCA* model
	*BRCA1* (n=323)	*BRCA2* (n=309)	*BRCA* (n=338)
**Threshold, %**	6.6	4	12.2
**Sensitivity, %**	69 (66-73)	67 (61-73)	73 (71-75)
**Specificity, %**	68 (67-68)	67 (66-67)	73 (72-73)
**Eligible for genetic testing, %**	35 (30-40)	35 (30-40)	33 (28-38)
**Detection ratio**	6:1		3:1
*Total number of patients screened, n (%)*	178 (53)		111 (33)
*Total number of carriers identified, n (%)*	30 (68)		32 (73)
*Total number of carriers missed, n (%)*	14 (32)		12 (27)

Sample: 338 ovarian cancer patients from the Malaysian Ovarian Cancer Genetic (OVC) study and the Mainstreaming Genetic Counselling for Ovarian Cancer Patients in Malaysia (MaGiC) study in imputed validation set.

Note: Variables included in BRCA-specific models (Model 2 and 3): Age of diagnosis, ethnicity, oral contraceptive use, menopausal status, parity status, family history of breast or ovarian cancer (first and second degree), personal history of cancer (ovarian, breast, or colorectal cancer), tumor grade, cancer stage and subtype. Variables included in Overall BRCA model (Model 3): Age of diagnosis, ethnicity, family history of breast or ovarian cancer (first and second degree), personal history of cancer (ovarian, breast, or colorectal cancer), laterality, tumor grade, cancer stage and subtype.

**Table 4:** Comparison of performance across different *BRCA* carrier status assessment methods

Risk assessment method	Overall *BRCA* model		Universal testing
**Threshold**	12.2^a^	1^b^	-
**Sensitivity (%)**	73	100	100
**Accuracy (%)**	77	26	13
**Eligible (%)**	33	85	100
**Detection ratio**	3:1	6:1	8:1
**Total cost of genetic testing (USD)**	150,000	386,364	454,545
**Genetic testing cost ratio (USD per detected carrier)**	1,974:1	3,715:1	4,371:1

Sample: 338 ovarian cancer patients from the Malaysian Ovarian Cancer Genetic (MyOvCa) study and the Mainstreaming Genetic Counselling for Ovarian Cancer Patients in Malaysia (MaGiC) study in imputed validation set.

a Optimal threshold.

b Threshold at 100% consistent with the standard clinical practice.

Note: Total cost of genetic testing (~USD570 per test) and corresponding cost per carrier detected were calculated based on country-specific annual incidence (800 cases) and mutation prevalence (13%), assuming 100% uptake and compliance in high-risk and low-risk groups, respectively. Variables included in Overall BRCA model (Model 3): Age of diagnosis, ethnicity, family history of breast or ovarian cancer (first and second degree), personal history of cancer (ovarian, breast, or colorectal cancer), laterality, tumor grade, cancer stage and subtype.

## Data Availability

The data generated in this study are not publicly available because they contain sensitive patient information and are subject to ethical data protection requirements, but are available from the corresponding author on reasonable request and with appropriate approvals.
