# Supplementary material for: BRCA testing in Asian ovarian cancer patients: Standard clinical practice or Mutation prediction model?
Source: Cancer Epidemiol Biomarkers Prev. Author manuscript; Available in PMC 2026 Jul 23. (PMC7619263; doi:10.1158/1055-9965.EPI-25-2008)
Supplement: Fig. S1 [file EMS215447-supplement-Fig__S1.docx]

# SUPPLEMENTAL MATERIALS

# FIGURE LEGENDS

**Supplementary Fig. S1.** Study design and sample selection

*Sample: Ovarian cancer patients from the Malaysian Ovarian Cancer Genetic (MyOvCa) study and the Mainstreaming Genetic Counselling for Ovarian Cancer Patients in Malaysia (MaGiC) study.*

##
