# Supplementary material for: BRCA testing in Asian ovarian cancer patients: Standard clinical practice or Mutation prediction model?
Source: Cancer Epidemiol Biomarkers Prev. Author manuscript; Available in PMC 2026 Jul 23. (PMC7619263; doi:10.1158/1055-9965.EPI-25-2008)
Supplement: Fig. S2 [file EMS215447-supplement-Fig__S2.docx]

# SUPPLEMENTAL MATERIALS

# FIGURE LEGENDS

**Supplementary Fig. S2.** Selection of optimal thresholds of best performing models

*Sample: 338 ovarian cancer patients from the Malaysian Ovarian Cancer Genetic (MyOvCa) study and the Mainstreaming Genetic Counselling for Ovarian Cancer Patients in Malaysia (MaGiC) study in imputed validation set.*

*Note: Variables included in BRCA-specific models (Model 2 and 3): Age of diagnosis, ethnicity, oral contraceptive use, menopausal status, parity status, family history of breast or ovarian cancer (first and second degree), personal history of cancer (ovarian, breast, or colorectal cancer), tumor grade, cancer stage and subtype. Variables included in Overall BRCA model (Model 3): Age of diagnosis, ethnicity, family history of breast or ovarian cancer (first and second degree), personal history of cancer (ovarian, breast, or colorectal cancer), laterality, tumor grade, cancer stage and subtype.*

##
