# Supplementary material for: BRCA testing in Asian ovarian cancer patients: Standard clinical practice or Mutation prediction model?
Source: Cancer Epidemiol Biomarkers Prev. Author manuscript; Available in PMC 2026 Jul 23. (PMC7619263; doi:10.1158/1055-9965.EPI-25-2008)

# SUPPLEMENTAL MATERIALS

# FIGURE LEGENDS

**Supplementary Fig. S3.** Screening rate and the corresponding total cost of genetic testing based on annual ovarian cancer incidence at varying sensitivity under reduced unit test cost

*Sample: 338 ovarian cancer patients from the Malaysian Ovarian Cancer Genetic (MyOvCa) study and the Mainstreaming Genetic Counselling for Ovarian Cancer Patients in Malaysia (MaGiC) study in imputed validation set.*

*Note: Total cost of genetic testing (~USD115 per test) was calculated based on country-specific annual incidence (800 cases), assuming 100% uptake and compliance in high-risk and low-risk groups, respectively.*


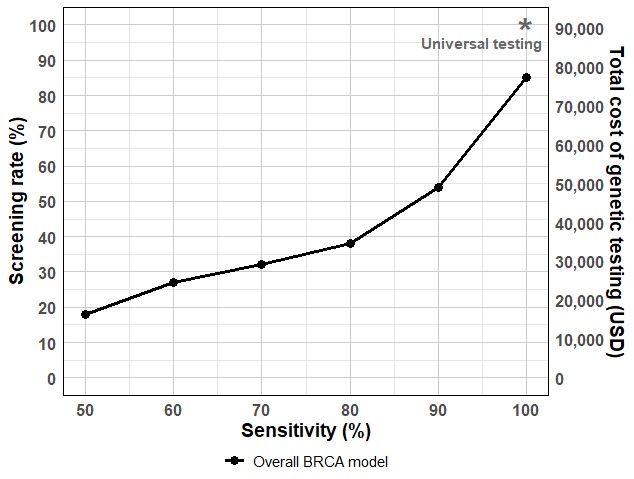

Supplement: Fig. S3 [file EMS215447-supplement-Fig__S3.docx]
