# Supplementary material for: BRCA testing in Asian ovarian cancer patients: Standard clinical practice or Mutation prediction model?
Source: Cancer Epidemiol Biomarkers Prev. Author manuscript; Available in PMC 2026 Jul 23. (PMC7619263; doi:10.1158/1055-9965.EPI-25-2008)

# SUPPLEMENTAL MATERIALS

# FIGURE LEGENDS

**Supplementary Fig. S4.** Screening rate and the corresponding total cost of genetic testing based on annual ovarian cancer incidence at varying subsidy level

*Sample: 338 ovarian cancer patients from the Malaysian Ovarian Cancer Genetic (OVC) study and the Mainstreaming Genetic Counselling for Ovarian Cancer Patients in Malaysia (MaGiC) study in imputed validation set.*

*Note: Screening rate (100% for Universal testing and 85% for Overall BRCA model) was adjusted for expected uptake based on subsidy level (34% uptake for 50% subsidy; 48% uptake for 80% subsidy; 92% uptake for 100% subsidy). Total cost of genetic testing was calculated based on annual incidence (800 cases) and adjusted screening rate, assuming 100% compliance in low-risk group.*


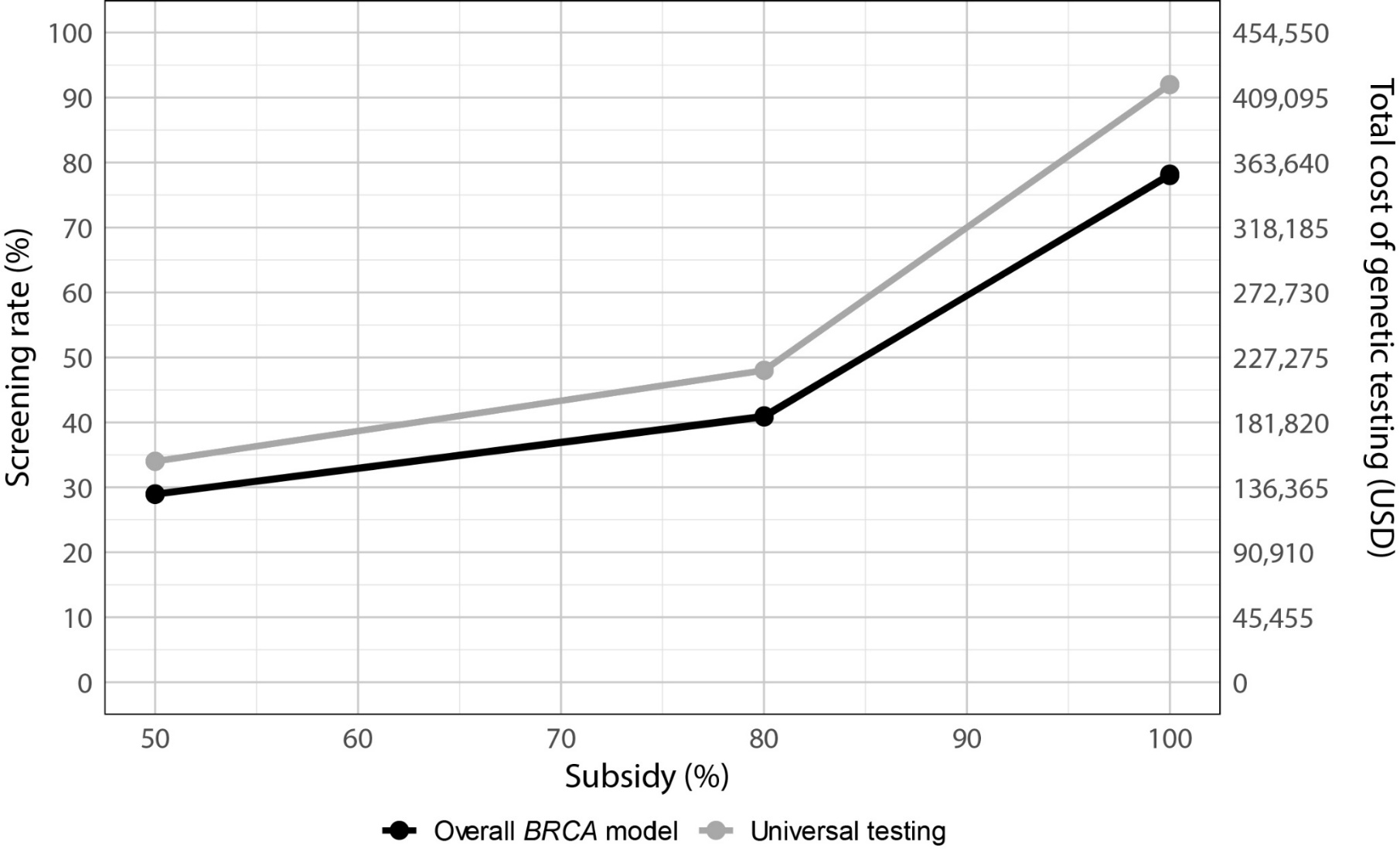

Supplement: Fig. S4 [file EMS215447-supplement-Fig__S4.docx]
