# Supplementary material for: BRCA testing in Asian ovarian cancer patients: Standard clinical practice or Mutation prediction model?
Source: Cancer Epidemiol Biomarkers Prev. Author manuscript; Available in PMC 2026 Jul 23. (PMC7619263; doi:10.1158/1055-9965.EPI-25-2008)
Supplement: Table S1 [file EMS215447-supplement-Table_S1.docx]

# SUPPLEMENTAL MATERIALS

## Supplementary Table S1. Distribution of variables by training and validation set

| **Variable** | **Training set**  **n (%)**  **(n=788)** | **Validation set**  **n (%)**  **(n=388)** | **P-value** |
| --- | --- | --- | --- |
| **Demographic** |  |  |  |
| **Age at diagnosis,** *mean (sd)* | 51.84 (11.2) | 51.83 (11.6) | 0.990 |
| **Age at consent,** *mean (sd)* | 52.58 (11.3) | 52.67 (11.5) | 0.906 |
| **Ethnicity,** *n (%)* |  |  | 0.139 |
| Chinese | 325 (41.5) | 164 (48.5) |  |
| Malay | 328 (41.8) | 122 (36.1) |  |
| Indian | 80 (10.2) | 35 (10.4) |  |
| Other | 51 (6.5) | 17 (5.0) |  |
| **Hormonal use and reproductive history** |  |  |  |
| **Oral contraceptive,** *n (%)* |  |  | 0.370 |
| Never | 617 (79.2) | 255 (76.6) |  |
| Ever | 162 (20.8) | 78 (23.4) |  |
| **Age at menarche,** *mean (sd)* | 12.99 (1.5) | 13.09 (1.6) | 0.301 |
| **Menopausal status,** *n (%)* |  |  | 0.646 |
| Pre-menopause | 152 (19.7) | 61 (18.3) |  |
| Post-menopause | 621 (80.3) | 273 (81.7) |  |
| **Parity status,** *n (%)* |  |  | 0.567 |
| Nulliparous | 261 (33.4) | 106 (31.5) |  |
| Parous | 520 (66.6) | 231 (68.5) |  |
| **Tubal Ligation,** *n (%)* |  |  | 0.090 |
| Never | 684 (90.8) | 279 (87.2) |  |
| Ever | 69 (9.2) | 41 (12.8) |  |
| **Breastfeeding,** *n (%)* |  |  | 0.915 |
| Never | 125 (24.9) | 53 (24.2) |  |
| Ever | 377 (75.1) | 166 (75.8) |  |
| **Hormone receptor therapy,** *n (%)* |  |  | 0.146 |
| Never | 568 (94.7) | 234 (91.8) |  |
| Ever | 32 (5.3) | 21 (8.2) |  |
| **Family history** |  |  |  |
| **FFHBC,** *n (%)* |  |  | 0.857 |
| No | 704 (90.5) | 305 (91.0) |  |
| Yes | 74 (9.5) | 30 (9.0) |  |
| **Age of FFHBC,** *mean (sd)* | 46.52 (12.96) | 45.91 (10.81) | 0.841 |
| **FFHOC,** *n (%)* |  |  | 0.862 |
| No | 741 (95.4) | 317 (94.9) |  |
| Yes | 36 (4.6) | 17 (5.1) |  |
| **Age of FFHOC,** *mean (sd)* | 55.59 (13.61) | 54.69 (14.90) | 0.834 |
| **SFHBC,** *n (%)* |  |  | 0.925 |
| No | 717 (92.2) | 310 (92.5) |  |
| Yes | 61 (7.8) | 25 (7.5) |  |
| **SFHOC,** *n (%)* |  |  | 0.577 |
| No | 766 (98.6) | 328 (97.9) |  |
| Yes | 11 (1.4) | 7 (2.1) |  |
| **Personal history** |  |  |  |
| **Type of cancer,** *n (%)* |  |  |  |
| Ovarian | 742 (95.0) | 324 (95.9) | 0.831 |
| Fallopian tube | 15 (1.9) | 6 (1.8) | 0.621 |
| Peritoneal | 24 (3.1) | 8 (2.4) | 0.162 |
| **Other cancer,** *n (%)* |  |  |  |
| Breast cancer | 36 (76.6) | 12 (60.0) | 0.539 |
| Uterine cancer | 8 (17.0) | 6 (30.0) | 0.447 |
| Cervical cancer | 3 (6.4) | 2 (10.0) | >0.999 |
| Colorectal cancer | 3 (6.4) | 2 (10.0) | >0.999 |
| **Tumour characteristics** |  |  |  |
| **Laterality,** *n (%)* |  |  | 0.594 |
| Unilateral | 369 (64.7) | 155 (62.5) |  |
| Bilateral | 201 (35.3) | 93 (37.5) |  |
| **Grade,** *n (%)* |  |  | 0.133 |
| Grade 1 | 60 (11.4) | 18 (7.9) |  |
| Grade 2 | 4 (0.8) | 0 (0.0) |  |
| Grade 3 | 461 (87.8) | 211 (92.1) |  |
| **Stage,** *n (%)* |  |  | 0.811 |
| Stage 1 | 194 (29.4) | 80 (28.6) |  |
| Stage 2 | 90 (13.6) | 38 (13.6) |  |
| Stage 3 | 310 (47.0) | 128 (45.7) |  |
| Stage 4 | 66 (10.0) | 34 (12.1) |  |
| **Subtype,** *n (%)* |  |  | 0.944 |
| Serous | 389 (50.9) | 176 (53.5) |  |
| Endometrioid | 157 (20.5) | 68 (20.7) |  |
| Clear cell | 134 (17.5) | 50 (15.2) |  |
| Mucinous | 27 (3.5) | 13 (4.0) |  |
| Mixed | 18 (2.4) | 7 (2.1) |  |
| Adenocarcinoma | 11 (1.4) | 3 (0.9) |  |
| Rare/Unclassified | 28 (3.7) | 12 (3.6) |  |
| **Outcome** |  |  |  |
| ***BRCA* PVs carrier status,** *n (%)* |  |  | >0.999 |
| Non-carrier | 685 (86.9) | 294 (87.0) |  |
| *BRCA1* | 68 (8.6) | 29 (8.6) |  |
| *BRCA2* | 35 (4.4) | 15 (4.4) |  |
| *Sample:1,126 ovarian cancer patients from the Malaysian Ovarian Cancer Genetic (OVC) study and the Mainstreaming Genetic Counselling for Ovarian Cancer Patients in Malaysia (MaGiC) study before imputation.*  *Abbreviations: FFHBC, First Degree Family History for Breast Cancer; FFHOC, First Degree Family History for Ovarian Cancer; SFHBC, Second Degree Family History for Breast Cancer; SFHOC, Second Degree Family History for Ovarian Cancer; PV, pathogenic variant.* | | | |
