# Supplementary material for: BRCA testing in Asian ovarian cancer patients: Standard clinical practice or Mutation prediction model?
Source: Cancer Epidemiol Biomarkers Prev. Author manuscript; Available in PMC 2026 Jul 23. (PMC7619263; doi:10.1158/1055-9965.EPI-25-2008)
Supplement: Table S2 [file EMS215447-supplement-Table_S2.docx]

# SUPPLEMENTAL MATERIALS

## Supplementary Table S2. Comparison of *BRCA* PVs carrier status

| **Variable** | ***BRCA1*** | **Non-carrier** | **P-value** | ***BRCA2*** | **Non-carrier** | **P-value** |
| --- | --- | --- | --- | --- | --- | --- |
|  | **n (%)** | **n (%)** |  | **n (%)** | **n (%)** |  |
|  | **(n=97)** | **(n=979)** |  | **(n=50)** | **(n=979)** |  |
| **Demographic** |  |  |  |  |  |  |
| **Age at diagnosis,** *mean (sd)* | 50.19 (10.4) | 51.84 (11.5) | 0.142 | 55.02 (8.4) | 51.84 (11.5) | **0.014** |
| **Ethnicity,** *n (%)* |  |  | 0.056 |  |  | 0.098 |
| Chinese | 32 (33.0) | 443 (45.4) |  | 14 (28.0) | 443 (45.4) |  |
| Malay | 49 (50.5) | 375 (38.5) |  | 26 (52.0) | 375 (38.5) |  |
| Indian | 12 (12.4) | 96 (9.8) |  | 7 (14.0) | 96 (9.8) |  |
| Other | 4 (4.1) | 61 (6.3) |  | 3 (6.0) | 61 (6.3) |  |
| **Hormonal use and reproductive history** |  |  |  |  |  |  |
| **Oral contraceptive,** *n (%)* |  |  | 0.294 |  |  | 0.606 |
| Never | 80 (83.3) | 755 (78.2) |  | 37 (74.0) | 755 (78.2) |  |
| Ever | 16 (16.7) | 211 (21.8) |  | 13 (26.0) | 211 (21.8) |  |
| **Age at menarche,** *mean (sd)* | 13.02 (1.5) | 13.02 (1.5) | 0.790 | 12.98 (1.6) | 13.02 (1.5) | 0.863 |
| **Menopausal status,** *n (%)* |  |  | 0.722 |  |  | **0.004** |
| Pre-menopause | 17 (18.1) | 195 (20.2) |  | 1 (2.1) | 195 (20.2) |  |
| Post-menopause | 77 (81.9) | 770 (79.8) |  | 47 (97.9) | 770 (79.8) |  |
| **Parity status,** *n (%)* |  |  | 0.459 |  |  | **0.002** |
| Nulliparous | 29 (29.9) | 332 (34.2) |  | 6 (12.0) | 332 (34.2) |  |
| Parous | 68 (70.1) | 639 (65.8) |  | 44 (88.0) | 639 (65.8) |  |
| **Tubal Ligation,** *n (%)* |  |  | 0.523 |  |  | 0.242 |
| Never | 80 (87.0) | 836 (89.7) |  | 47 (95.9) | 836 (89.7) |  |
| Ever | 12 (13.0) | 96 (10.3) |  | 2 (4.1) | 96 (10.3) |  |
| **Family history** |  |  |  |  |  |  |
| **FFHBC,** *n (%)* |  |  | **<0.001** |  |  | **<0.001** |
| No | 67 (69.8) | 906 (93.6) |  | 36 (73.5) | 906 (93.6) |  |
| Yes | 29 (30.2) | 62 (6.4) |  | 13 (26.5) | 62 (6.4) |  |
| **FFHOC,** *n (%)* |  |  | **<0.001** |  |  | 0.483 |
| No | 75 (79.8) | 937 (96.8) |  | 46 (93.9) | 937 (96.8) |  |
| Yes | 19 (20.2) | 31 (3.2) |  | 3 (6.1) | 31 (3.2) |  |
| **SFHBC,** *n (%)* |  |  | **0.025** |  |  | **0.024** |
| No | 83 (86.5) | 903 (93.3) |  | 41 (83.7) | 903 (93.3) |  |
| Yes | 13 (13.5) | 65 (6.7) |  | 8 (16.3) | 65 (6.7) |  |
| **SFHOC,** *n (%)* |  |  | **0.001** |  |  | 0.915 |
| No | 90 (93.8) | 955 (98.8) |  | 49 (100.0) | 955 (98.8) |  |
| Yes | 6 (6.3) | 12 (1.2) |  | 0 (0.0) | 12 (1.2) |  |
| **Personal history** |  |  |  |  |  |  |
| **Type of cancer,** *n (%)* |  |  |  |  |  |  |
| Ovarian | 91 (94.8) | 930 (95.5) | **<0.001** | 45 (91.8) | 930 (95.5) | **0.005** |
| Fallopian tube | 3 (3.1) | 15 (1.5) | 0.784 | 3 (6.1) | 15 (1.5) | 0.784 |
| Peritoneal | 2 (2.1) | 29 (3.0) | 0.924 | 1 (2.1) | 29 (3.0) | 0.961 |
| **Other cancer,** *n (%)* |  |  |  |  |  |  |
| Breast cancer | 13 (100.0) | 29 (56.9) | **<0.001** | 6 (75.0) | 29 (56.9) | **0.002** |
| Uterine cancer | 0 (0.0) | 14 (27.5) | 0.474 | 0 (0.0) | 14 (27.5) | 0.822 |
| Cervical cancer | 0 (0.0) | 5 (9.8) | >0.999 | 0 (0.0) | 5 (9.8) | >0.999 |
| Colorectal cancer | 0 (0.0) | 3 (5.9) | >0.999 | 2 (25.0) | 3 (5.9) | **0.009** |
| **Tumor characteristics** |  |  |  |  |  |  |
| **Laterality,** *n (%)* |  |  | 0.666 |  |  | 0.056 |
| Unilateral | 40 (61.5) | 469 (65.0) |  | 15 (46.9) | 469 (65.0) |  |
| Bilateral | 25 (38.5) | 252 (35.0) |  | 17 (53.1) | 252 (35.0) |  |
| **Grade,** *n (%)* |  |  | **0.005** |  |  | **0.009** |
| Grade 1 | 0 (0.0) | 78 (12.0) |  | 0 (0.0) | 78 (12.0) |  |
| Grade 2 | 1 (1.6) | 2 (0.3) |  | 1 (2.6) | 2 (0.3) |  |
| Grade 3 | 63 (98.4) | 571 (87.7) |  | 38 (97.4) | 571 (87.7) |  |
| **Stage,** *n (%)* |  |  | **<0.001** |  |  | **<0.001** |
| Stage 1 | 7 (8.6) | 263 (32.3) |  | 4 (8.9) | 263 (32.3) |  |
| Stage 2 | 9 (11.1) | 116 (14.3) |  | 3 (6.7) | 116 (14.3) |  |
| Stage 3 | 49 (60.5) | 358 (44.0) |  | 31 (68.9) | 358 (44.0) |  |
| Stage 4 | 16 (19.8) | 77 (9.4) |  | 7 (15.6) | 77 (9.5) |  |
| **Subtype,** *n (%)* |  |  | **<0.001** |  |  | **<0.001** |
| Serous | 75 (82.4) | 452 (47.4) |  | 38 (77.6) | 452 (47.4) |  |
| Endometrioid | 11 (12.1) | 209 (21.9) |  | 5 (10.2) | 209 (21.9) |  |
| Clear cell | 0 (0.0) | 181 (19.0) |  | 3 (6.1) | 181 (19.0) |  |
| Mucinous | 0 (0.0) | 25 (2.6) |  | 0 (0.0) | 25 (2.6) |  |
| Mixed | 1 (1.1) | 12 (1.3) |  | 1 (2.0) | 12 (1.3) |  |
| Adenocarcinoma | 3 (3.3) | 35 (3.7) |  | 2 (4.1) | 35 (3.7) |  |
| Rare/Unclassified | 1 (1.1) | 39 (4.1) |  | 0 (0.0) | 39 (4.1) |  |
| *Sample: 1,126 ovarian cancer patients from the Malaysian Ovarian Cancer Genetic (OVC) study and the* *Mainstreaming Genetic Counselling for Ovarian Cancer Patients in Malaysia (MaGiC) study before imputation.* | | | | | | |
| *Abbreviations: FFHBC, First Degree Family History for Breast Cancer; FFHOC, First Degree Family History for Ovarian Cancer; SFHBC, Second Degree Family History for Breast Cancer; SFHOC, First Degree Family History for Ovarian Cancer.* | | | | | | |
