# Supplementary material for: BRCA testing in Asian ovarian cancer patients: Standard clinical practice or Mutation prediction model?
Source: Cancer Epidemiol Biomarkers Prev. Author manuscript; Available in PMC 2026 Jul 23. (PMC7619263; doi:10.1158/1055-9965.EPI-25-2008)
Supplement: Table S3 [file EMS215447-supplement-Table_S3.docx]

# SUPPLEMENTAL MATERIALS

## Supplementary Table S3. Univariable regression by *BRCA* PVs carrier status

| **Variable** | ***BRCA1 versus* Non-carrier (n=753)** | | | | ***BRCA2* versus Non-carrier (n=720)** | | | | ***BRCA* versus Non-carrier (n=788)** | | | |
| --- | --- | --- | --- | --- | --- | --- | --- | --- | --- | --- | --- | --- |
|  | **OR** | **95% CI** | | **P-value** | **OR** | **95% CI** | | **P-value** | **OR** | **95% CI** | | **P-value** |
| **Demographic** |  |  |  |  |  |  |  |  |  |  |  |  |
| **Age at diagnosis** | 0.99 | 0.97 | 1.01 | 0.426 | 1.03 | 0.99 | 1.06 | **0.113** | 1.00 | 0.98 | 1.02 | 0.795 |
| **Ethnicity** |  |  |  |  |  |  |  |  |  |  |  |  |
| Chinese | *Reference* | |  |  | *Reference* | |  |  | *Reference* | |  |  |
| Malay | 1.42 | 0.82 | 2.45 | 0.206 | 3.39 | 1.42 | 8.06 | **0.006** | 1.85 | 1.16 | 2.95 | **0.010** |
| Indian | 1.40 | 0.61 | 3.25 | 0.428 | 3.13 | 0.96 | 10.20 | **0.058** | 1.78 | 0.89 | 3.58 | **0.105** |
| Other | 0.49 | 0.11 | 2.14 | 0.342 | 0.87 | 0.10 | 7.30 | 0.902 | 0.57 | 0.17 | 1.95 | 0.374 |
| **Hormonal use and reproductive history** | |  |  |  |  |  |  |  |  |  |  |  |
| **Oral contraceptive** |  |  |  |  |  |  |  |  |  |  |  |  |
| Never | *Reference* |  |  | *Reference* |  |  | *Reference* |  |  |  |  |  |
| Ever | 0.43 | 0.19 | 0.97 | **0.041** | 1.70 | 0.81 | 3.56 | **0.157** | 0.79 | 0.46 | 1.36 | 0.397 |
| **Age at menarche** | 1.01 | 0.86 | 1.20 | 0.868 | 1.02 | 0.81 | 1.28 | 0.861 | 1.02 | 0.88 | 1.17 | 0.818 |
| **Menopausal status** |  |  |  |  |  |  |  |  |  |  |  |  |
| Pre-menopause | *Reference* | |  |  | *Reference* | |  |  | *Reference* | |  |  |
| Post-menopause | 1.43 | 0.71 | 2.88 | 0.314 | 8.36 | 1.13 | 61.70 | **0.037** | 2.06 | 1.07 | 3.96 | **0.030** |
| **Parity status** |  |  |  |  |  |  |  |  |  |  |  |  |
| Nulliparous | *Reference* | |  |  | *Reference* | |  |  | *Reference* | |  |  |
| Parous | 0.99 | 0.58 | 1.66 | 0.958 | 5.74 | 1.74 | 18.97 | **0.004** | 1.51 | 0.95 | 2.42 | **0.082** |
| **Tubal Ligation** |  |  |  |  |  |  |  |  |  |  |  |  |
| Never | *Reference* | |  |  | *Reference* | |  |  | *Reference* | |  |  |
| Ever | 1.27 | 0.55 | 2.91 | 0.574 | 0.63 | 0.15 | 2.71 | 0.538 | 1.05 | 0.50 | 2.18 | 0.902 |
| **Family history** |  |  |  |  |  |  |  |  |  |  |  |  |
| **Family history of cancer** | *Reference (No)* | |  |  | *Reference (No)* | |  |  | *Reference (No)* | | |  |
| FFHBC (yes) | 6.38 | 3.57 | 11.39 | **<0.001** | 5.00 | 2.27 | 11.00 | **<0.001** | 5.89 | 3.56 | 9.75 | **<0.001** |
| FFHOC (yes) | 7.31 | 3.70 | 14.47 | **<0.001** | 2.20 | 0.62 | 7.81 | 0.223 | 5.37 | 2.85 | 10.10 | **<0.001** |
| SFHBC (yes) | 2.42 | 1.16 | 5.05 | **0.019** | 2.36 | 0.87 | 6.39 | **0.091** | 2.40 | 1.28 | 4.48 | **0.006** |
| SFHOC (yes) | 5.98 | 1.70 | 21.01 | **0.005** | - | - | - | - | 3.91 | 1.12 | 13.59 | **0.032** |
| **Personal history** |  |  |  |  |  |  |  |  |  |  |  |  |
| **Type of cancer** |  |  |  |  |  |  |  |  |  |  |  |  |
| Ovarian | *Reference* | |  |  | *Reference* | |  |  | *Reference* | |  |  |
| Fallopian tube | 3.14 | 0.84 | 11.70 | **0.088** | 4.05 | 0.85 | 19.30 | **0.079** | 3.45 | 1.16 | 10.31 | **0.027** |
| Peritoneal | 0.49 | 0.07 | 3.66 | 0.485 | 0.90 | 0.12 | 6.92 | 0.920 | 0.63 | 0.15 | 2.71 | 0.534 |
| **Other cancer** | *Reference (No)* | |  |  | *Reference (No)* | |  |  | *Reference (No)* | | |  |
| Breast cancer (yes) | 7.13 | 3.31 | 15.34 | **<0.001** | 4.29 | 1.38 | 13.33 | **0.012** | 6.11 | 3.05 | 12.26 | **<0.001** |
| Uterine cancer (yes) | - | - | - | - | - | - | - | - | - | - | - | - |
| Cervical cancer (yes) | - | - | - | - | - | - | - | - | - | - | - | - |
| Colorectal cancer (yes) | - | - | - | - | 10.04 | 0.89 | 113.99 | **0.063** | 3.35 | 0.30 | 37.40 | 0.326 |
| **Tumor characteristics** |  |  |  |  |  |  |  |  |  |  |  |  |
| **Laterality** |  |  |  |  |  |  |  |  |  |  |  |  |
| Unilateral | *Reference* | |  |  | *Reference* | |  |  | *Reference* | |  |  |
| Bilateral | 0.90 | 0.49 | 1.64 | 0.727 | 0.47 | 0.21 | 1.08 | **0.077** | 0.72 | 0.44 | 1.18 | **0.193** |
| **Grade** |  |  |  |  |  |  |  |  |  |  |  |  |
| Grade 1-2 | *Reference* | |  |  | *Reference* | |  |  | *Reference* | |  |  |
| Grade 3 | 5.00 | 1.17 | 21.39 | **0.030** | 4.15 | 0.65 | 26.60 | **0.132** | 4.48 | 1.49 | 13.49 | **0.008** |
| **Stage** |  |  |  |  |  |  |  |  |  |  |  |  |
| Stage 1 | *Reference* | |  |  | *Reference* | |  |  | *Reference* | |  |  |
| Stage 2 | 4.50 | 1.31 | 15.52 | **0.017** | - | - | - | - | 2.54 | 0.89 | 7.28 | **0.081** |
| Stage 3 | 6.18 | 2.14 | 17.84 | **0.001** | 5.81 | 1.74 | 19.47 | **0.004** | 5.99 | 2.66 | 13.47 | **<0.001** |
| Stage 4 | 10.84 | 3.36 | 34.95 | **<0.001** | 3.27 | 0.64 | 16.61 | **0.153** | 7.54 | 2.93 | 19.43 | **<0.001** |
| **Subtype** |  |  |  |  |  |  |  |  |  |  |  |  |
| Other ^a^ | *Reference* | |  |  | *Reference* | |  |  | *Reference* | |  |  |
| Mucinous | 0.72 | 0.07 | 7.17 | 0.776 | - | - | - | - | 0.36 | 0.04 | 3.14 | 0.355 |
| Clear cell | - | - | - | - | 0.25 | 0.04 | 1.57 | **0.141** | 0.13 | 0.02 | 0.66 | **0.014** |
| Endometrioid | 0.96 | 0.25 | 3.77 | 0.958 | 0.47 | 0.10 | 2.18 | 0.335 | 0.71 | 0.25 | 2.01 | 0.523 |
| Serous | 2.88 | 0.86 | 9.60 | **0.085** | 1.31 | 0.38 | 4.52 | 0.664 | 2.09 | 0.86 | 5.06 | **0.103** |
| **Subtype-Grade v1** |  |  |  |  |  |  |  |  |  |  |  |  |
| Other ^a^ | *Reference* | |  |  | *Reference* | |  |  | *Reference* | |  |  |
| Mucinous | 0.72 | 0.07 | 7.17 | 0.776 | - | - | - | - | 0.36 | 0.04 | 3.14 | 0.355 |
| Clear cell | - | - | - | - | 0.25 | 0.04 | 1.57 | **0.141** | 0.13 | 0.02 | 0.66 | **0.014** |
| Endometrioid | 0.96 | 0.25 | 3.77 | 0.958 | 0.47 | 0.10 | 2.18 | 0.335 | 0.71 | 0.25 | 2.01 | 0.523 |
| Low grade serous | - | - | - | - | - | - | - | - | - | - | - | - |
| High grade serous | 3.22 | 0.96 | 10.74 | **0.058** | 1.47 | 0.43 | 5.06 | 0.540 | 2.33 | 0.96 | 5.66 | **0.061** |
| **Subtype-Grade v2** |  |  |  |  |  |  |  |  |  |  |  |  |
| Other ^b^ | *Reference* | |  |  | *Reference* | |  |  | *Reference* | |  |  |
| High grade clear cell | - | - | - | - | 0.68 | 0.11 | 4.15 | 0.676 | 0.28 | 0.06 | 1.40 | **0.122** |
| High grade endometrioid | 2.65 | 0.76 | 9.20 | **0.125** | 1.67 | 0.33 | 8.49 | 0.533 | 2.23 | 0.83 | 5.98 | **0.113** |
| High grade serous | 6.16 | 2.22 | 17.13 | **0.001** | 3.91 | 1.17 | 13.06 | **0.027** | 5.16 | 2.35 | 11.37 | **<0.001** |
| *Sample: 788 ovarian cancer patients from the Malaysian Ovarian Cancer Genetic (OVC) study and the Mainstreaming Genetic Counselling for Ovarian Cancer Patients in Malaysia (MaGiC) study in imputed training set.*  *Abbreviations: OR, Odds ratio; 95% CI, 95% Confidence Interval; FFHBC, First Degree Family History for Breast Cancer; FFHOC, First Degree Family History for Ovarian Cancer; SFHBC, Second Degree Family History for Breast Cancer; SFHOC, First Degree Family History of Ovarian Cancer.* | | | | | | | | | | | | |
| *^a^ Includes mixed, adenocarcinoma, rare, and unclassified.* | | | |  |  |  |  |  |  |  |  |  |
| *^b^ Includes mixed, adenocarcinoma, rare, unclassified, mucinous, low-grade clear cell, low-grade endometrioid, and low-grade* | | | | | | | | | | | |  |
| *serous.* |  |  |  |  |  |  |  |  |  |  |  |  |
