# Supplementary material for: BRCA testing in Asian ovarian cancer patients: Standard clinical practice or Mutation prediction model?
Source: Cancer Epidemiol Biomarkers Prev. Author manuscript; Available in PMC 2026 Jul 23. (PMC7619263; doi:10.1158/1055-9965.EPI-25-2008)
Supplement: Table S4 [file EMS215447-supplement-Table_S4.docx]

# SUPPLEMENTAL MATERIALS

## Supplementary Table S4. Performance of multivariable regression models by *BRCA* PVs carrier status

| **Model** | ***BRCA1 versus* Non-carrier (n=323)** | | ***BRCA2* versus Non-carrier (n=309)** | | ***BRCA* versus Non-carrier (n=338)** | |
| --- | --- | --- | --- | --- | --- | --- |
|  | **AUC (95% CI)** | **HL (P-value)** | **AUC (95% Cl)** | **HL (P-value)** | **AUC (95% Cl)** | **HL (P-value)** |
| **Baseline model** |  |  |  |  |  |  |
| Model 1 | 0.72 (0.61-0.82) | 7.3 (0.502) | 0.68 (0.54-0.82) | 6.6 (0.593) | 0.71 (0.62-0.79) | 5.7 (0.697) |
| **Reproductive model** |  |  |  |  |  |  |
| Model 2 | 0.69 (0.58-0.79) | 13.9 (0.096) | **0.72 (0.59-0.84)** | **15.0 (0.115)** | 0.74 (0.66-0.81) | 11.8 (0.189) |
| **Subtype model** |  |  |  |  |  |  |
| Model 3 | **0.80 (0.72-0.87)** | **13.8 (0.834)** | 0.67 (0.51-0.82) | >100 (>0.999) | **0.80 (0.74-0.87)** | **8.7 (0.608)** |
| Model 4 | 0.80 (0.72-0.88) | >100 (>0.999) | 0.68 (0.52-0.83) | >100 (0.090) | 0.80 (0.73-0.87) | 60.7 (>0.999) |
| Model 5 | 0.81 (0.73-0.88) | 20.7 (>0.999) | 0.67 (0.51-0.83) | >100 (>0.999) | 0.81 (0.74-0.87) | 13.3 (0.472) |
| **Full model** |  |  |  |  |  |  |
| Model 6 | 0.78 (0.70-0.86) | 23.7 (0.268) | 0.68 (0.52-0.84) | >100 (>0.999) | 0.80 (0.74-0.87) | 12.3 (0.482) |
| Model 7 | 0.78 (0.70-0.86) | >100 (>0.999) | 0.69 (0.54-0.85) | >100 (0.146) | 0.80 (0.74-0.88) | 54.9 (>0.999) |
| Model 8 | 0.78 (0.71-0.86) | 29.4 (0.948) | 0.68 (0.52-0.85) | >100 (>0.999) | 0.80 (0.75-0.88) | 16.9 (0.680) |
| *Sample: 338 ovarian cancer patients from the Malaysian Ovarian Cancer Genetic (OVC) study and the Mainstreaming Genetic Counselling for Ovarian Cancer Patients in Malaysia (MaGiC) study in imputed validation set.* | | | | | | |
| *Abbreviations: AUC, Area Under Curve; 95% CI, 95% Confidence Interval; HL, Hosmer-Lemeshow.* | | | | | | |
| *Note: Model 3 and 6 (grade and subtype as independent variables); Model 4 and 7 (Subtype-Grade v1); Model 5 and 8 (Subtype-Grade v2).* | | | | | | |
